# Decoding the mechanisms of amyloid-β in synaptic toxicity

**DOI:** 10.1177/13872877261424292

**Published:** 2026-03-13

**Authors:** Piotr Toruński, Giusy Pizzirusso, Bengt Winblad, Luis Enrique Arroyo-García

**Affiliations:** 1 Division of Neurogeriatrics, Department of Neurobiology, Care Sciences and Society, 27106 Karolinska Institutet, Solna, Sweden; 2 Department of Women's and Children's Health, 27106 Karolinska Institutet, Solna, Sweden; 3 Theme Inflammation and Aging, Karolinska University Hospital, Huddinge, Sweden

**Keywords:** Alzheimer's disease, amyloid-β, excitotoxicity, neuronal function, oscillatory activity, synaptic plasticity

## Abstract

Amyloid-β (Aβ) aggregation is considered a central hallmark in the pathophysiology of Alzheimer's disease (AD). Aβ protein aggregates disrupt synaptic architecture, calcium homeostasis, and mitochondrial function, leading to excitotoxicity and synaptic plasticity deficits. Animal models and human studies reveal that Aβ-induced alterations in synaptic activity and neuronal circuit function appear before irreversible damage and the onset of cognitive impairment. This review examines the multifaceted effects of Aβ on synaptic and neuronal circuits across its distinct aggregation states, including monomeric, oligomeric, protofibrillar, and fibrillar forms. Its novelty lies in providing a comprehensive map of Aβ-induced mechanisms that disrupt neuronal electrical function, based on electrophysiological evidence from neuronal cultures, animal models, and patient studies, with a particular focus on preclinical stages of cognitive decline. We suggest that the Aβ-induced synaptic toxicity could serve, first, as a complementary biomarker of brain deterioration and second, as a readout of current AD therapies. This could potentially lead to better outcomes in AD treatments.

## Introduction

Alzheimer's disease (AD) is widely known by its progressive cognitive decline, memory impairment, and eventually, loss of independence.^1^ The neuropathological features of AD include the accumulation of two misfolded proteins, amyloid-β (Aβ) and hyperphosphorylated microtubule-associated tau, both of which contribute to neuronal dysfunction and neurodegeneration.^1^ According to the amyloid hypothesis, the aggregation of Aβ peptides, particularly in the form of oligomers and plaques, is responsible for triggering downstream events, including tau pathology, synaptic dysfunction, neuroinflammation, and neuronal loss, ultimately leading to cognitive decline.^2,3^ Other, revised versions of the hypothesis also consider disease variants of AD, together with environmental and genetic factors.^4^ Aβ-related changes are likely the earliest pathological feature in AD onset, which progress slowly and appear even 18 years before a diagnosis is made, making Aβ a primary target for understanding the disorder at stages most suitable for intervention.^5,6^

After over a century since AD was first described, the only disease-modifying treatments approved by the US Food and Drug Administration (FDA) target Aβ accumulation, reflecting its central role in the pathology of the disorder.^7,8^ These treatments, specifically lecanemab and donanemab, have shown an effective clearance of Aβ deposits in the brain together with moderate effects in slowing cognitive decline, as assessed in trials involving patients in advanced stages of AD (mild cognitive impairment (MCI) and mild-dementia AD).^9–11^ Recent advancements in blood-based biomarkers, particularly those using measures of phosphorylated tau alone or in combination with Aβ, now allow for early and non-invasive detection of Aβ pathology with accuracy comparable to cerebrospinal fluid (CSF) tests.^12–14^ This enables the testing of anti-amyloid treatments at even earlier disease stages, where intervention may yield more favorable outcomes.^10,11^ Nevertheless, it is imperative to understand the effects of toxic Aβ on neuronal function during the early stages of the amyloidogenic pathology progression. This, combined with early biomarker detection and the development of new biomarkers for neuronal dysfunction, prior to structural damage and the onset of cognitive impairment, could facilitate preventive treatment strategies, shifting AD management toward early intervention rather than late-stage symptom treatment.

Aβ is a heterogeneous peptide that affects neurophysiology differently depending on the exact species, aggregation state, and concentration.^15–18^ This variability complicates our ability to generalize findings and fully comprehend the mechanisms through which Aβ contributes to AD pathology. This review aims to explore recent advancements in our understanding of the neurotoxic effects of Aβ, with particular focus on the peptide's heterogeneity. Its novelty lies in providing a comprehensive map of Aβ-induced mechanisms that disrupt neuronal electrical function, based on electrophysiological evidence from neuronal cultures, animal models, and patient studies, with a particular focus on preclinical stages of cognitive decline. We aim to describe its diverse effects in pathological conditions, first to explain the potential damage of toxic Aβ in the brain, and second to guide the research field to develop functional biomarkers that aid in the treatment of AD in early stages.

## Aβ production and aggregation

Aβ is a toxic and aggregation-prone polypeptide consisting of 30–51 amino acid residues, generated through the proteolysis of amyloid-β protein precursor (AβPP), a highly conserved transmembrane protein found in the brains of various animals, including humans, monkeys, dogs, and mice.^19–21^ AβPP is a type I transmembrane protein with a large, glycosylated extracellular N-terminus, a single membrane-spanning domain, and a shorter cytoplasmic C-terminus.^16^ The production and release of Aβ occurs through the amyloidogenic pathway that involves the sequential cleavage of AβPP by the enzymes β-secretase 1 (BACE1) and γ-secretase.^22,23^ The proteolytic γ-secretase cleavage creates two main Aβ species, Aβ_1−40_ and Aβ_1−42_, which will be the focus of the review.^16^

Several therapeutic approaches have focused on the amyloidogenic pathway as a treatment for AD. These strategies focused on inhibiting BACE1 and γ-secretase, with several drugs entering clinical trials only to be halted due to reported side effects.^24^ A new promising therapeutic target involves modulation of γ-secretase instead of inhibiting the enzyme, an approach that does not dysregulate cellular pathways the same way as the previous and thus should not produce serious side effects.^25,26^

### Aggregation of Aβ and creation of toxic peptides

Aβ peptide can be found in a variety of aggregates formed of Aβ monomers that result in oligomers, protofibrils, and amyloid fibrils that can present themselves in the form of plaques.^16^ The pathological conversion of monomers into other aggregates marks a critical step in Aβ's role from physiological to toxic. This shift can be aggravated by changes in AβPP processing, oxidative stress, mitochondrial dysfunction, and changes in the local ionic microenvironment (e.g., elevated levels of zinc and copper), all of which can induce Aβ misfolding and promote β-sheet formation.^27–30^ Additionally, the failure of proteostasis networks, ranging from protein synthesis to protein degradation and insufficient clearance of Aβ, facilitates the assembly of monomers into oligomers.^18^

Among Aβ peptides, Aβ_1−40_ is the most prevalent, constituting approximately 90% of the total Aβ produced in the brain, with Aβ_1−42_ accounting for near the remaining 10% of Aβ aggregates.^31^ Aβ_1−40_ is more soluble and less prone to aggregation compared to longer Aβ peptides.^32^ Conversely, Aβ_1−42_ is more hydrophobic due to its two additional amino acids at the C-terminus, making it highly prone to aggregation and forming oligomers and fibrils.^33^ These properties render Aβ_1−42_ more neurotoxic and more closely associated with the formation of amyloid plaques found in AD brains.^34^

Protein glycation has been identified as an important step in the development of higher aggregate states of Aβ.^35^ Depositions of Aβ usually consist of glycated proteins, and glycated Aβ has been found to accelerate the aggregation of soluble Aβ in vitro as compared with non-glycated Aβ.^36^ N-terminal truncations of Aβ are less soluble, more prone to aggregation, and associated with enhanced toxicity.^37^ In particular, pyroglutamylated variants form when N-terminal truncations expose a glutamate residue, which is then transformed into pyroglutamate by the enzyme glutaminyl cyclase, with pyroglutamylated Aβ considered more cytotoxic and prone to aggregation in mouse hippocampal slices.^38^ By contrast, the oxidation of methionine 35 reduces the aggregation propensity of the peptide by reducing both hydrophobic and electrostatic association in vitro.^39^ Moreover, longer C-terminal variants such as Aβ_1−43_ have been implicated in seeding aggregation of other Aβ peptides and in neurotoxicity.^40,41^

## Monomers: from physiology to pathology

**Figure 1.**
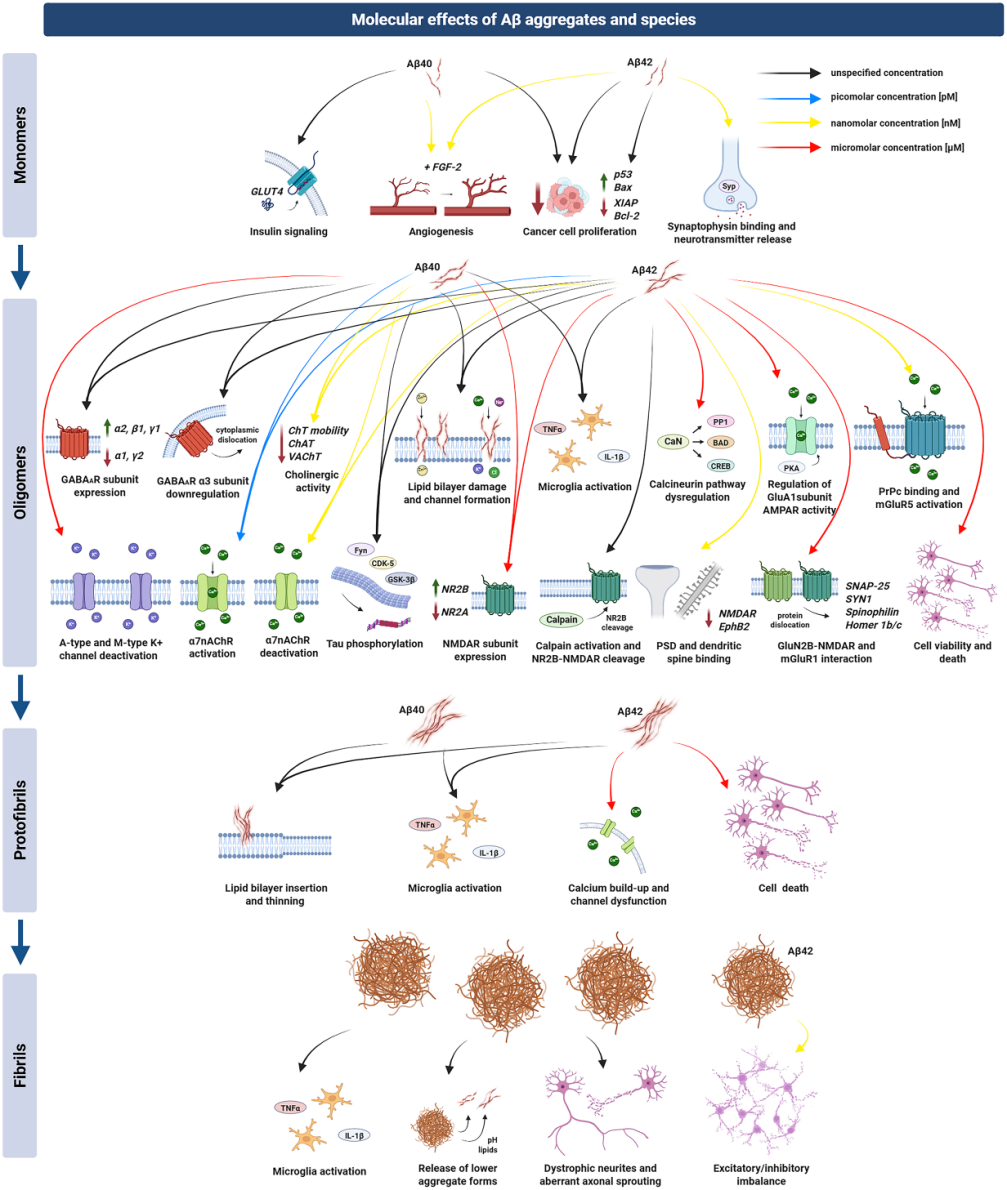
Molecular effects of Aβ aggregates and species. **Monomeric Aβ** modulates several molecular pathways related to synaptic function, cell proliferation, and angiogenesis. These monomers affect insulin signaling (e.g., GLUT4) and vascular growth (e.g., FGF-2), while influencing apoptosis- or survival-related proteins (p53, Bax, XIAP, Bcl-2). They can also bind presynaptic proteins, thereby impacting neurotransmitter release. This binding possibly explains why nanomolar Aβ_1−42_ decreases the slope of field excitatory postsynaptic potentials (fEPSPs) in the CA3/CA1 hippocampal region under theta-burst stimulation (TBS) protocols, thus impairing LTP.^47^ **Oligomeric Aβ** exerts more extensive effects on neuronal and glial physiology, spanning picomolar to micromolar concentrations. The figure shows that oligomers engage α7-nicotinic acetylcholine receptors, GABA_A_ receptors, voltage-gated potassium channels, and NMDA/AMPA receptor complexes, leading to cholinergic dysfunction, tau phosphorylation, and calcium homeostasis dysregulation. Oligomers also trigger microglial activation and can induce excitotoxicity by disturbing glutamate reuptake. These mechanisms of action underly the effects found in cell functional activity. Micromolar Aβ_1−40_ increases the firing variability of cholinergic and GABAergic cells and augments firing in glutamatergic cells.^48^ At picomolar levels, Aβ_1−42_ enhances presynaptic neurotransmitter release, boosting miniature EPSC frequency and docked synaptic vesicles in CA1 neurons.^49^ Oligomers at nanomolar concentrations desynchronize hippocampal CA3 pyramidal neurons,^50^ while micromolar doses desynchronize CA3 fast-spiking interneurons from gamma oscillations.^51^ Additionally, human-derived mixed-species Aβ oligomers that interact with NR2B-containing NMDA receptors reduce fEPSP slopes in CA3/CA1 LTP protocols by inhibiting glutamate reuptake.^52^ Aβ oligomers are potent disruptors of excitability and synaptic synchronization. **Protofibrillar Aβ** can insert into membranes, cause lipid bilayer thinning, and foster microglial activation. The figure shows that protofibrils facilitate calcium influx and partial disassembly into smaller toxic aggregates. Electrophysiological data show that nanomolar Aβ_1−40_ elevates the frequency of excitatory postsynaptic currents and action potentials in neocortical cultured neurons.^53^ At micromolar concentrations, Aβ_1−42_ decreases neuronal input resistance and increases resting membrane potential in SH-SY5Y cells and reduces the fEPSP slope in CA3/CA1 LTP protocols in hippocampal slices.^54^ Moreover, nanomolar concentration of dimers in which serine 26 was substituted with cysteine (Aβ_S26C_) blocks TBS-LTP in CA3/CA1 hippocampal slices.^55^ In this way, Aβ protofibrils are significant modulators of membrane properties and synaptic plasticity. **Fibrillar Aβ** aggregates are shown in the figure to induce microglial activation, release lower-order aggregates, and promote dystrophic neurites. They typically exert more pronounced structural and inflammatory consequences rather than direct receptor or channel modulation. However, artificial but humanized Aβ_1−42_ fibrils can impair the gamma oscillatory activity of mouse hippocampal slices in the ex vivo configuration.^50,56^ Similarly, low nanomolar concentrations of Aβ_1−42_ fibrils extracted from the App^NL−G−F^ and App^NL−F^ mouse models impair the gamma oscillations in hippocampal slices of wild-type mice.^57^

Aβ monomers are generally not considered toxic and appear to play significant physiological roles, including angiogenesis, cancer suppression, and insulin signalling.^42,43^ A visual summary of the molecular effects of Aβ monomers related to electrophysiological functions is presented in Figure 1. Both Aβ_1−40_ and Aβ_1−42_ at nanomolar concentrations synergize with fibroblast growth factor-2 (FGF-2), a major regulator of endothelial cell function, to promote blood vessel formation.^44^ Studies using zebrafish embryos lacking AβPP have demonstrated that monomeric Aβ_1−42_ restores vascular abnormalities and increases vascular branching in a dose-dependent manner, underscoring its pro-angiogenic properties.^45,46^

In vitro studies show that both monomeric Aβ_1−40_ and Aβ_1−42_ reduce the growth of human glioblastoma, breast cancer, and melanoma cells when injected into tumor-bearing mice or on cancer cell lines, slowing tumor progression.^58,59^ Interestingly, intracellular Aβ_1−42_ enhances the activity of p53 in samples from AD-affected human brains.^60^ P53 is a crucial regulator of apoptosis, indicating that even at higher than physiological concentrations, Aβ can regulate cancer growth through programmed cell death.^61^ Furthermore, monomeric Aβ_1−40_ also contributes to the regulation of insulin signaling. It facilitates glucose homeostasis by phosphorylating enzymes involved in insulin signaling pathways and promoting the translocation of glucose transporter 4 (GLUT4) to the cell membrane in cell cultures.^62^ Aβ_1−40_ further acts as a negative modulator of insulin secretion in peripheral tissues in a mouse model, linking it to glucose and insulin metabolism.^63^

Specific Aβ_1−42_ monomers affect synaptic activity at different levels. In rat hippocampal CA3–CA1 neurons, Aβ_1−42_ monomers at nanomolar concentrations localize to presynaptic terminals, where they compete with vesicle-associated membrane protein 2 (VAMP2) for binding to synaptophysin (Syp).^64^ This competition facilitates the formation of the fusion pore complex, leading to an increase in primed synaptic vesicles and enhanced neurotransmitter release.^64^ However, nanomolar levels of Aβ_1−42_ impair synaptic plasticity as demonstrated by the blocking of long-term potentiation (LTP) by theta burst stimulation in the CA1 area of the hippocampus, while the same concentration of monomeric Aβ_1−40_ does not affect it.^47^ Furthermore, previous work from our lab has shown that nanomolar concentrations of Aβ_1−42_ have a modest effect on ex vivo hippocampal gamma oscillations.^50,65^ These findings suggest that Aβ_1−42_ monomers in slightly high concentrations (but still at nanomolar levels) have an impact on synaptic function that could start affecting memory and learning circuits in early stages of the AD pathology progression.

## Oligomers: key agents of neurotoxicity

Aβ oligomers (AβOs) are defined as Aβ assemblies that are not pelleted from physiological fluids by high-speed centrifugation.^66^ Mass spectrometry studies have shown that brain-derived bioactive 7 kDa AβOs are composed of a heterogeneous mixture of covalently cross-linked dimers of Aβ between 37 and 42 residues long.^67,68^ AβOs are considered the smallest neurotoxic form of Aβ due to their soluble, diffusible nature and high affinity for synaptic sites.^16^ Even though AβOs are more toxic compared to monomers, they also exhibit physiological roles in antimicrobial defense, brain injury recovery, cancer suppression, and synaptic modulation, depending on their concentration and aggregation state.^42^ The molecular effects and interactions of AβOs related to electrophysiological functions are visualized in Figure 1.

AβOs can bind to microbial cell walls through their heparin-binding domain, inducing agglutination and preventing pathogen adhesion to host cells in transgenic 5xFAD mice.^69^ Additionally, oligomeric Aβ disrupts microbial membranes by forming toxic ion channels that cause uncontrolled ion movement and subsequent pathogen death.^70^ In traumatic brain injury models, injecting oligomeric Aβ_1−40_ improves motor recovery and memory function, highlighting its neuroprotective role.^71^ Similarly, reducing endogenous Aβ in mice hinders recovery in brain and spinal cord injury models, suggesting a protective mechanism.^72^ Similarly to monomers, AβOs also appear to suppress cancer progression, as both human Aβ_1−40_ and Aβ_1−42_ oligomers, when injected in mice transplanted with human glioblastoma and lung cancer cells, significantly slowed tumor progression.^59^ However, oligomeric Aβ affects various neuronal functions and brain circuit mechanisms, which are described in detail below.

### Aβos, effects, and mechanisms of function

Aβ oligomers have been shown to affect neuronal and synaptic function, plasticity mechanisms, membrane structure, ion balance, and inflammatory activation.^73–75^ This makes a complex mechanism of AβOs that needs to be fully understood to comprehend its impact on the brain.

AβOs are suggested to stimulate microglial activation through tyrosine kinase-dependent pathways, leading to increased protein phospho-tyrosine levels and the release of tumor necrosis factor-alpha (TNF-α), a key pro-inflammatory cytokine that exacerbates neuroinflammation and neuronal damage in microglia cell cultures.^76^ Additionally, AβOs drive the activation of the nucleotide-binding oligomerization domain-like receptor family pyrin domain-containing 3 (NLRP3) inflammasome, which leads to caspase-1 activation and the subsequent processing of interleukin-1 beta (IL-1β), further amplifying neuroinflammation and microglial toxicity in neuron-microglia co-cultures.^77^ Both low molecular weight (LMW) AβOs and protofibrils strongly induce IL-1β release and promote the formation of apoptosis-associated speck-like protein containing caspase recruitment domain (ASC) specks in microglia in cell cultures.^78^ These findings indicate that AβOs contribute to the microglial-mediated neuroinflammation that leads to dysfunction, before fibrillar Aβ deposition occurs.

Literature shows that AβOs can affect the neuronal ion balance in several ways. Both Aβ_1−40_ and Aβ_1−42_ oligomers can disrupt the lipid bilayer through direct membrane insertion and ion channel formation, affecting ion conductance in lipid membranes.^79–82^ These ion channels can be both non-selective or selective for specific ions, with the literature identifying Zn^2+−^sensitive channels formed by Aβ_1−40_ and Aβ_1−42_ in neuronal cultures and artificial lipid bilayers.^83,84^ However, under closer to physiological conditions, only Aβ_1−42_ oligomers formed ion-conducting pores in neuronal cultures.^85^ The conductance of Aβ_1−42_ induced channels is stronger when using membranes with brain-extracted lipids, highlighting the role of ion channel formation by Aβ peptides in physiological conditions.^86^ It has been demonstrated that Aβ-induced perforations in neuronal membranes cause excessive Ca^2+^ influx and synaptic failure in cell cultures.^87,88^ Another study reported that small-molecule blockers of these Aβ-induced channels effectively protect neurons from cytotoxicity in cell cultures.^89^ A pentapeptide derived from Aβ's glycine zipper region was shown to inhibit channel formation and prevent Ca^2+^ dysregulation in hippocampal primary cultures.^90^ Additionally, increased intraneuronal calcium levels exacerbate excitotoxicity and mitochondrial stress.^91^ AβO-induced calcium overload triggers the opening of the mitochondrial permeability transition pore (mPTP), leading to cellular energy deficits and initiating apoptosis or necrosis, further compounding neuronal stress.^92,93^

The ability of Aβ_1−42_ oligomers to disrupt cellular membranes works through a carpeting process where AβOs are inserted into the upper leaflet of the membrane in vitro.^94^ This interaction increases membrane conductance by causing lateral spreading of phospholipid head groups, a phenomenon often described as membrane thinning.^95^ Furthermore, Aβ_1−42_ oligomers can interact with lipid bilayers by extracting lipids in a detergent-like manner in vitro^96^​. This extraction process allows lipids to associate with the growing fibrils, consistent with the high lipid content observed in Aβ plaques, adding to the aggregation propensity of these species.^97,98^ AβOs increase intracellular calcium levels by disrupting the lipid bilayer, impacting calcium pathways, and impacting neuronal signaling through cholinergic and glutamatergic systems in cell cultures.^99^

Both Aβ_1−40_ and Aβ_1−42_ oligomers at concentrations ranging from picomolar to micromolar have been found to contribute to synaptic dysfunction and excitotoxicity, a pathological process driven by excessive neuronal activity and calcium influx, through their interactions with synaptic receptors and proteins.^73–75^ Moreover, AβOs at nanomolar concentrations have shown impairment in LTP in the CA1 area of the hippocampus.^47^ However, the specific effect of AβOs on neuronal and synaptic functions depends on the neuronal subtype.

### Cholinergic system dysregulation

Oligomeric Aβ_1−42_ has multifaceted and concentration-dependent effects on synaptic activity. At picomolar concentrations, oligomeric Aβ_1−42_ activates subunit alpha 7 nicotinic acetylcholine receptors (α7 nAChRs) in CA1 hippocampal pyramidal neurons, leading to Ca^2+^ entry and increased neurotransmitter release.^49^ This initiates a cascade of intracellular events involving the nitric oxide (NO)-cyclic guanosine monophosphate (cGMP)-protein kinase G (PKG) pathway.^49^ Consequently, key plasticity-related molecules are activated, including phosphorylated cAMP response element-binding protein (p-CREB), phosphorylated calcium/calmodulin-dependent protein kinase II (p-CaMKII), and brain-derived neurotrophic factor (BDNF).^49^ As a result, picomolar concentrations of oligomeric Aβ_1−42_ enhance synaptic transmission and LTP in CA1 hippocampal pyramidal neurons by increasing presynaptic neurotransmitter release, the frequency of miniature excitatory post-synaptic currents, and the number of docked synaptic vesicles in mouse hippocampal slices.^47,49^ In contrast, at nanomolar concentrations, oligomeric Aβ_1−42_ impairs synaptic plasticity and memory by disrupting both presynaptic and postsynaptic mechanisms through an antagonist action on α7 nAChRs in mouse hippocampal slices and organotypic cultures.^47,49,100^ This dual effect highlights the importance of understanding Aβ concentration dynamics in physiological and pathological contexts.

AβOs have profound effects on neurotransmitter systems. Both Aβ_1−40_ and Aβ_1−42_ exhibit high picomolar affinity for α7nAChR, a pentameric cholinergic channel.^101^ Picomolar concentrations of Aβ_1−40_ introduced to ex vivo rat hippocampal preparations can activate α7nAChR, triggering pathways regulating synaptic plasticity, learning, and memory. However, nanomolar concentrations and prolonged exposure can desensitize and inactivate the receptor, causing synaptic disruption.^102,103^ This binding is linked to receptor internalization and the intracellular accumulation of Aβ in cell cultures and post-mortem human brain tissue.^104^ In vivo electrochemical recordings in young and aged rats indicate reduced depolarization-evoked cholinergic signals in aged rats infused with Aβ_1−42_ using osmotic minipumps.^105^ Additionally, young and aged Aβ-infused rats display an impaired ability of cholinergic synapses to remove exogenous choline from the extracellular space mediated by a reduced mobility of choline transporters and decreased expression of presynaptic cholinergic proteins (choline acetyltransferase and vesicular acetylcholine transporter) required for acetylcholine synthesis and release.^105^

This inability to remove exogenous choline induces sustained increases in presynaptic calcium release via α7-containing and non-α7-containing nAChRs, in synaptosomes from rat hippocampi, striata, and cortices, contributing to synaptic dysregulation.^106^ Moreover, low nanomolar concentrations of Aβ_1−42_ significantly inhibit choline acetyltransferase activity in cultured cholinergic neurons, an effect mediated by oxidative stress and disrupted by glutamate receptor antagonists or antioxidants.^107^ Interestingly, cholinergic dysfunction in patients with AD precedes neuronal loss, indicating involvement in the early stages of the pathology.^107^ In AD patients, the number of nAChRs negatively correlates with the levels of AβOs, particularly total oligomers in early-onset AD and decamers in late-onset AD.^108^ This reduction in nAChRs underlies synaptic and network dysfunction, highlighting the widespread influence of AβOs on neuronal pathways.

Specific cholinergic neuronal populations also exhibit altered excitability in response to Aβ_1−42_. For instance, basal forebrain cholinergic neurons (BFCNs) exposed to nanomolar concentrations of oligomeric Aβ_1−42_ demonstrate increased intrinsic excitability, reduced afterhyperpolarization, and altered action potential kinetics in cell cultures.^109^ These changes are mediated through subunit alpha 7 and subunit beta 2 nicotinic acetylcholine receptors, as evidenced by improved spatial memory in genetically modified APP/PS1 mice lacking this receptor subunit.^109^ Findings on the exposure of BFCNs to Aβ_1−42_ bear special significance, as the cholinergic system of the basal forebrain is one of the first regions affected in AD and plays a critical role in modulating cognitive functions such as attention, learning, and memory.^110–113^ 5xFAD mice show decreased neuronal excitability and enhanced afterhyperpolarization in CA1 neurons in mouse hippocampal slices, an effect dependent on muscarinic acetylcholine receptors and correlated with an increase in brain levels of Aβ_1−42._^114^ Together, these findings underscore the multifaceted mechanisms by which Aβ modulates the cholinergic pathway, with visible physiological functions that, due to accumulation and disruption in Aβ clearance, cause disruption to nicotinic transmission.

### Glutamatergic system dysregulation

AβOs can also dysregulate the function of the glutamatergic system through several pathways. At high micromolar concentrations, Aβ_1−40_ oligomers increase the firing of glutamatergic cells by blocking A-type and M-type potassium channels and contributing to excitotoxicity.^48^ Micromolar oligomeric Aβ_1−40_ and Aβ_1−42_ affect synaptic function by suppressing NR2A-containing NMDA receptors and activating NR2B-containing NMDA receptors, which lead to downstream activation of caspase-3 and caspase-8 in rat hippocampal neuronal primary cultures, establishing a link between excitotoxicity, synaptic dysfunction, and apoptotic pathways.^115^ NMDA receptors are also involved in modulating the effect that AβOs have on synaptic proteins, as when antagonists to NMDA receptors are used, AβOs do not affect postsynaptic density-95 levels, a synaptic scaffolding protein.^115^ The downstream consequences of these disruptions exacerbate already present calcium dysregulation and extend to mitochondrial dysfunction.^99,116^

Interaction with NR2B-containing NMDA receptors in ex vivo hippocampal slices is also found when using mixed AβOs produced by neuronal cultures and AβOs derived from individuals with AD.^52^ Here, AβOs act similarly to a glutamate reuptake inhibitor, leading to impaired synaptic plasticity by inhibiting LTP in the Shaffer collateral-CA1 pathway in mouse hippocampal slices.^52^ The observed reduced glutamate reuptake leads to excessive activation of these extrasynaptic receptors, triggering downstream activation of calpain and phosphorylation of p38 mitogen-activated protein kinase and dephosphorylation of both cAMP response element-binding protein (CREB) and extracellular signal-regulated kinase.^52^

Aβ_1−42_ oligomers at micromolar concentrations exert additional effects on neuronal and synaptic integrity through interactions with specific glutamate receptors, particularly GluN2B-containing NMDA receptors and mGluR1 in primary neuronal cultures from the cortex.^117^ These interactions induce pathological synaptic responses, leading to the dislocation of key synaptic proteins, such as presynaptic SNAP-25, synapsin I, postsynaptic spinophilin, and homer 1b/c, without altering their overall protein levels.^117^ Micromolar Aβ_1−42_ oligomers also impact glutamatergic transmission through the GluA1 subunit of AMPA receptors in rat hippocampal slices and organotypic cultures.^118^ This effect is a response of neurons to intracellular Aβ and leads to a rapid increase in AMPA receptor activity and is visualized by an increase in the amplitude of AMPA-mediated excitatory postsynaptic currents.^118^

Furthermore, oligomeric Aβ_1−42_ at nanomolar concentrations impairs the excitatory/inhibitory equilibrium by desynchronizing the action potentials of CA3 pyramidal neurons in ex vivo hippocampal preparations, leading to a significant degradation in the power of gamma oscillations (20–80 Hz), which are critical for information integration and cognitive processes.^50,119,120^ Interestingly, hippocampal neurons lacking GluA3 are resistant to synaptic weakening and inhibition of synaptic plasticity induced by Aβ in acute and organotypic hippocampal slices, highlighting the involvement of AMPA receptors in Aβ pathology.^121^

The toxic effects of AβOs are not limited to functional disruptions; they extend to structural damage as well. Soluble Aβ_1−42_ oligomers at nanomolar concentrations have been shown to induce synapse loss in hippocampal cultures, particularly through binding to the postsynaptic density and dendritic spines.^73,122^ This synapse loss is associated with AβOs between 50 and 100 kDa and involves a decrease in the expression of NMDA and EphB2 receptors that leads to abnormal spine morphology and, in turn, reduced spine density.^73^ Additionally, AβOs reduce dendritic spine density by disrupting the actin cytoskeleton and associated synaptic proteins, impairing synaptic connectivity.^123^ In cell cultures, exposure to micromolar concentrations of Aβ_1−42_ oligomers has also been linked to decreased cell viability and induced cell death.^124^ This finding was later confirmed in in vitro and ex vivo rat brain slices, adding to the overall neuronal loss observed in AD.^125^

### GABAergic system dysregulation

GABAergic neurons are essential for the maintenance of the excitatory/inhibitory balance in the brain.^126^ Interestingly, recent findings show that GABAergic neurons appear to be particularly vulnerable to AβOs. The expression of GABA type A receptor (GABA_A_R) subunits α1 and γ2 is downregulated in the hippocampus of 5xFAD transgenic mice and cell membranes transplanted from AD patients, with subunits α2, β1, and γ1 being additionally upregulated in AD patients.^127,128^ Furthermore, studies have reported that Aβ pathology leads to a reduction in the number of GABAergic interneurons, namely somatostatin, neuropeptide Y, parvalbumin, and calretinin positive interneurons in transgenic mouse models and parvalbumin and calretinin positive interneurons in AD postmortem brain specimens.^129–133^ Similarly, in the locus coeruleus of APP/PS1 mice and AD patients, AβOs accumulates intraneuronally in noradrenergic neurons and in proximity to postsynaptic α3 subunit-containing GABA_A_Rs (α3-GABA_A_Rs).^134^ The immunoreactivity of α3-GABA_A_Rs shifts from these postsynaptic surfaces to cytoplasm, reducing cluster density, size, intensity, and membrane coverage, highlighting an effect on α3-GABA_A_Rs expression and function.^134^ In APP/PS1 mice, this coincided with locus coeruleus neuronal hyperexcitability, selective postsynaptic impairment of tonic inhibition, marked by elevated spontaneous firing rates and reduced noradrenaline levels locally, alongside heightened hippocampal noradrenaline.^134^

Several studies used patch-clamp electrophysiology to analyze the effects of AβOs on GABAergic transmission in ex vivo mouse brain slices. After exposing cortical layer V pyramidal neurons in ex vivo brain slices to micromolar concentrations of Aβ_1−42_, the function of GABA_A_Rs is downregulated, resulting in diminished inhibitory synaptic currents.^135^ Aβ_1−40_ oligomers at micromolar concentrations were found to affect the variability of firing of cholinergic and GABAergic cells in the medial septum.^48^ Analogously, we have shown that Aβ_1−42_ oligomers at micromolar concentrations impair the firing and excitatory/inhibitory balance of inhibitory interneurons in ex vivo hippocampus of wild-type mice.^51^ Moreover, using the *App^NL−G−F^* mouse model at a stage with a high concentration of AβOs and during the onset of Aβ fibrilization, we found alterations in the intrinsic properties, excitability, firing synchronization, and reduced inhibitory input of GABAergic interneurons in the CA3 area of the hippocampus.^136,137^ Altogether, these findings suggest a functional alteration of GABAergic neurons during early stages of the Aβ pathology that leads to neurodegeneration and the excitatory/inhibitory imbalance in neuronal networks.

### Calcineurin and calpain activation

The effects of Aβ_1−42_ oligomers additionally affect the dysregulation of intracellular calcium pathways by activating two calcium-dependent enzymes critical to neuronal function, calcineurin (CaN) and calpain. Micromolar Aβ_1−42_ oligomers selectively induce CaN activity in neuronal cultures and rat brain slices, an effect not observed with Aβ monomers or fibrils.^125^ CaN hyperactivation reduces the phosphorylation of CREB and Bcl-2 associated agonist of cell death (BAD) proteins, impairing synaptic plasticity and promoting apoptosis.^125,138,139^ Specifically, decreased CREB phosphorylation disrupts CREB-dependent transcription necessary for LTP of the AMPA receptor subunit GluA1 in mouse hippocampi.^140^ CaN is also responsible for activating PP1-dependent dephosphorylation of CamKII, important for proper ion channel function and neurotransmission.^139,141^ Additionally, BAD dephosphorylation leads to apoptosis, exacerbating neuronal loss.^142^ Researchers observed increased pro-apoptotic activity of BAD in the frontal cortex and hippocampus of 5xFAD mice.^143^ Notably, in vivo evidence from Tg2576 mice indicates that CaN activity increases and CREB phosphorylation decreases before plaque formation, revealing an early role in AD pathology.^125^ Similarly to CaN, elevated calpain activity is observed early in postmortem tissues from AD patients, preceding tau phosphorylation, synaptic protein loss, and neurodegeneration.^144^ This early calpain activation cleaves key synaptic proteins, such as the NMDA receptor subunit NR2B, and gives rise to active NMDA receptor forms that further contribute to excitotoxicity.^144,145^ Together, these findings highlight how Aβ_1−42_ oligomers disrupt calcium signaling by hyperactivating calcineurin and calpain, setting off a cascade of synaptic dysfunction, excitotoxicity, and apoptosis that precedes hallmark AD pathology and contributes to early dysfunction.

### Aβos and the cellular prion protein

One of the proteins that Aβ_1−42_ oligomers show binding affinity to is the cellular prion protein (PrP^c^).^146^ PrP^c^, similar to AβPP, is a cell surface protein that resides in cholesterol-rich lipid rafts of the cell membrane that interacts with AβPP, making PrP^c^ part of its interactome.^147^ Interestingly, PrP^c^ is involved in the modulation of currents evoked by NMDA receptors with NR2D subunits.^148^ PrP^c^ knock-out mice show excitotoxicity through enhanced and prolonged glutamatergic activity in the hippocampus, both in vitro and in vivo.^148^ Additionally, AβO- PrP^c^ complexes activate intracellular Fyn kinase through a mechanism that requires mGluR5 in neuronal cultures.^149^ This interaction leads to an increase in intracellular calcium in Xenopus oocytes and neurons.^149^ This interaction of AβOs with PrP^c^ also affects the network activity, as when Aβ_1−42_ oligomers at nanomolar concentrations are presented to hippocampal ex vivo preparations, they inhibit theta burst stimulation LTP between hippocampal CA3 and CA1 pyramidal cells at the Schaffer collateral pathway in wild-type mice, but not in mice lacking PrP^c.^^150^ This highlights the importance of PrP^c^ as a mediator of synaptic deficits inflicted by Aβ_1−42_ oligomers.

## Protofibrils

Protofibrils are formed during the progression from oligomers to mature fibrils and exhibit a high degree of heterogeneity in their structure. They are defined as the soluble oligomeric aggregates of Aβ peptides appearing as a peak in the void volume (>75 kDa) of a size exclusion chromatography with a Superdex G75 column.^151,152^ Like other oligomers, they are considered toxic, with some researchers considering them the most toxic Aβ aggregation state.^18,153^ This position has led to the creation of the monoclonal antibody Lecanemab, which preferentially binds to Aβ protofibrils, as a treatment for AD.^9^ The molecular mechanics related to electrophysiological functions and the role of Aβ protofibrils in Aβ pathology are presented in Figure 1.

Aβ protofibrils are strongly associated with inflammation.^154^ Astrocytes and microglia have been shown to contribute to the extracellular spread of Aβ protofibrils through extracellular vesicles.^155,156^ Aβ_1−42_ protofibrils stimulate TNFα production and regulate IL-1β through several mechanisms, including Toll-like receptor/MyD88-mediated priming, activation of the NLRP3 inflammasome, and modulation of the IL-1β secretion process, collectively suggesting broad impacts of Aβ_1−42_ protofibrils on the innate immune system.^157,158^ Additionally, like other AβOs, protofibrils interact and insert within lipid bilayers through a carpeting process.^94^ Here, this process also leads to membrane thinning, increasing membrane roughness and membrane conductance, possibly through pore formation, as for other AβOs.^159^

Protofibrils have also been shown to induce electrophysiological alterations and neurotoxicity in rat cortical neurons, as demonstrated in in vitro studies using neuronal cultures containing glial cells.^53^ Nanomolar concentrations of Aβ_1−40_ protofibrils induced marked increases in both the frequency of excitatory postsynaptic currents and action potentials of cortical neurons, highlighting their potent impact on neuronal excitability and synaptic function. This effect was not visible for low molecular weight Aβ_1−40_ oligomers.^53^ Further, ex vivo experiments using hippocampal preparations revealed that Aβ protofibrils in which serine 26 was substituted with cysteine (Aβ_S26C_), blocked LTP at nanomolar concentrations, impairing the function of pathways for spatial-temporal pattern separation and learning.^55^ These findings emphasize the role of Aβ protofibrils in the synaptic dysfunction observed in Aβ-related pathology.

In SH-SY5Y cells, LMW and protofibrils of micromolar Aβ_1−42_ elevated intracellular calcium, but pre-incubation reduced calcium influx upon depolarization, with protofibrils exerting a stronger effect.^54^ Electrophysiologically, only protofibrils decreased input resistance and increased resting membrane potential in SH-SY5Y cells.^54^ In hippocampal CA1 neurons, they reduced the field EPSP slope under high-frequency LTP protocols, whereas LMW oligomers had no effect, highlighting the greater functional disruption caused by protofibrils of Aβ_1−42._^54^ Another mode of action by which protofibrils contribute to disease pathology is facilitating the nucleation process that leads to the formation of mature fibrils and plaques.^18^

All of these results indicate that both Aβ_1−42_ LMW oligomers and protofibrils disrupt neuronal function on several levels; however, Aβ_1−42_ protofibrils inflict their damage by dysregulating voltage-gated calcium channels, inducing oxidative stress, and dysregulating ion homeostasis, that leads to damage of membrane structures and in turn impaired functional neuronal activity. These findings are hard to compare with previous reports on the role of AβOs in neurotoxicity since studies usually do not discriminate between AβOs of different molecular weights, making it difficult to distinguish the role of Aβ conformational states of different sizes. The division of AβOs into LMW and protofibril peptides faces an issue as well, as the majority of peptides reported in the LMW group are monomers and dimers, two conformational states that have different effects on neurons than the more generally described AβOs that include peptide sizes from dimers to protofibrils.^18,42,54,73,160^

## Fibrils and plaques

Fibrils are highly ordered β-sheet structures where Aβ peptides assemble into β-sheets with β-strands perpendicularly oriented to the long axis of the fibril and stabilized by hydrogen bonds that are insoluble and resistant to proteolytic degradation.^161^ These aggregates deposit extracellularly to form Aβ plaques, which are a hallmark of AD.^162^ The aggregation of fibrils into plaques represents the culmination of the Aβ aggregation pathway. Although fibrils are less dynamic than AβOs and protofibrils, their presence signifies advanced disease stages and chronic damage. Figure 1 presents a visual summary of the role of Aβ plaques in pathology.

We have shown that artificial but humanized Aβ_1−42_ fibrils impair the gamma oscillatory activity of mouse hippocampal slices in the ex vivo configuration.^50,56^ Furthermore, we have shown that low nanomolar concentrations of Aβ_1−42_ fibrils extracted from the *App^NL−G−F^* and *App^NL−F^* mouse models impair the gamma oscillations in hippocampal slices of wild-type mice.^57^ These results suggest that Aβ fibrils exert an acute toxic effect that impacts neuronal networks, causing an excitatory/inhibitory imbalance.

The deposition of Aβ plaques contributes to a hostile microenvironment that affects surrounding neuronal and glial cells.^16,163,164^ Plaques can disrupt the function of neural networks by affecting the morphology of neurites, negatively impacting the speed of transmission between neurons.^165^ Plaque accumulation is associated with persistent activation of astrocytes and microglia, which release cytokines such as IL-1β, TNF-α, and IFN-γ.^18,166^ While these immune responses initially aim to clear plaques, chronic activation leads to neuroinflammation that exacerbates neurodegeneration.^18,163^

The indirect effects of fibrils and Aβ plaques on cognition are significant due to their impact on spike timing of neurons by disrupting neurite morphology, as shown in postmortem tissue from AD patients.^165^ The area surrounding plaques is often characterized by dystrophic neurites and aberrant axonal sprouting, which reflects the brain's attempt to compensate for the disrupted synaptic networks but ultimately contributes to network dysfunction.^165,167^ These dystrophic neurites contain aggregates of hyperphosphorylated tau and other cellular components, showcasing the role of cellular transport in Aβ pathology.^168,169^ Lastly, the environment surrounding plaques is characterized by increases in oxidative stress that, together with gliosis, further destabilize the brain's cellular environment.^170–172^

## Aβ pathology in transgenic rodent animal models

Research in animal models of AD has helped understand how endogenous Aβ pathology influences the network activity in the brain, with most of the studies employing Aβ overexpressing transgenic animal models such as APP/PS1, APP23/PS45, 5xFAD, 3xTg-AD, TgF344-AD, Tg2576 (APPSwe), or more recently *App^NL−F^* and *App^NL−G−F^*, with the latter being characterized by a highly increased spread of Aβ in the brain.^173^ The APP/PS1 model carries human mutations in both the APP and PSEN1 genes.^174^ The mutations of the APP23/PS45 mice also carry mutations to the APP and PSEN1 genes.^175^ The 5xFAD mouse model expresses five familial AD mutations—three in the APP gene (Swedish, Florida, and London mutations) and two in the PSEN1 gene.^176^ The 3xTg-AD mouse model carries mutations in APP, PSEN1, and an additional mutation leading to tau pathology.^177^ The Tg2576 mice carry the Swedish mutation in the APP gene.^178^ Both the *App^NL−F^* and *App^NL−G−F^* mouse models include knock-in of mutations to the APP gene, with both containing the Swedish and the Iberian mutation, and *App^NL−G−F^* additionally having the Arctic mutation.^179,180^ More recently, a rat model has been developed carrying the Swedish, the Iberian, and the Arctic mutations in the APP gene.^181^ Transgenic TgF344-AD rats carry the Swedish APP mutation, together with a PSEN1 gene mutation.^182^

**Figure 2.**
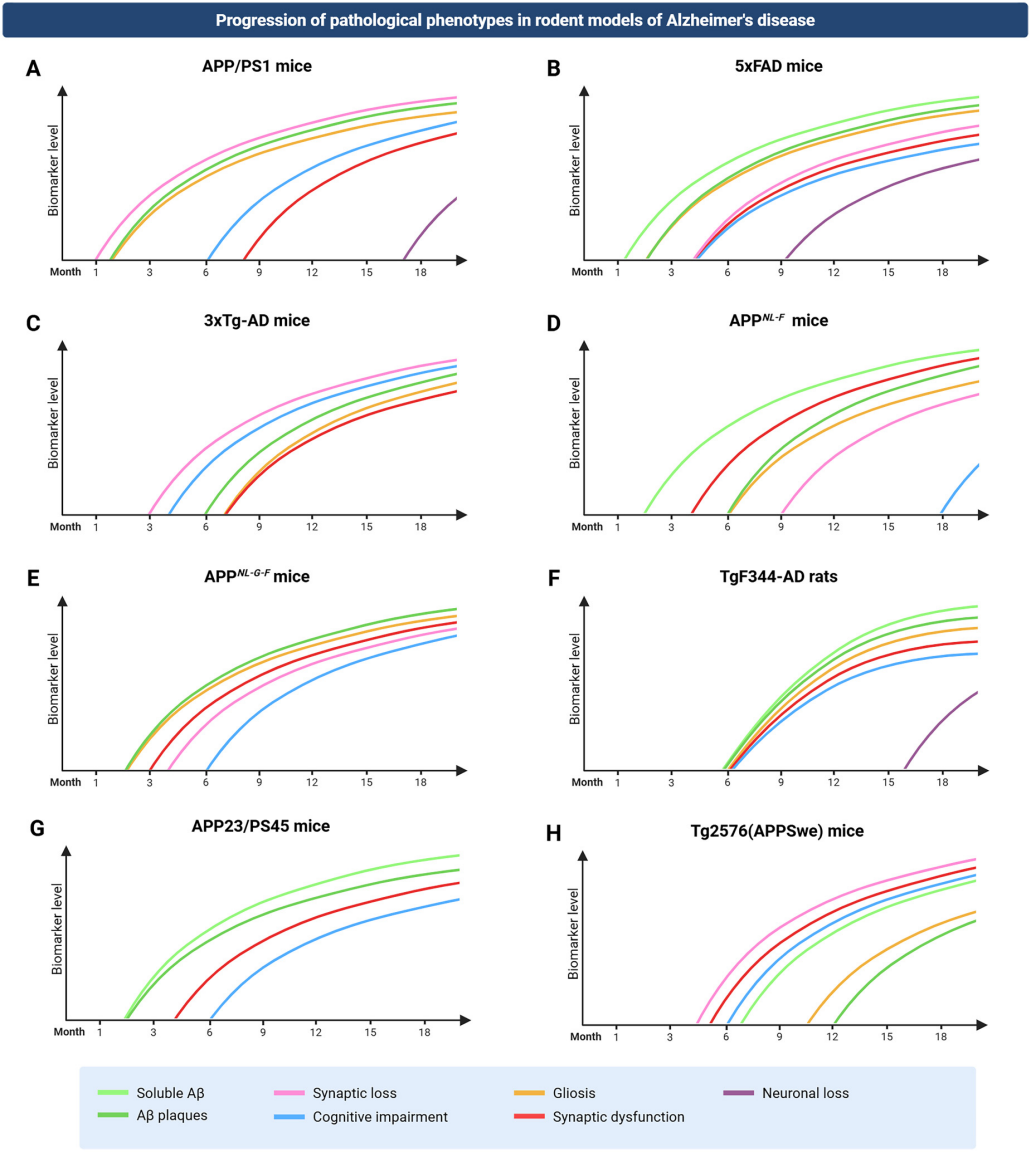
Progression of AD pathology phenotype as reported in several rodent models of AD. Timeline of the progression of the AD pathology phenotype for each mouse model is presented in Table 1.

The models described here represent a subset of the most widely used transgenic rodent lines that show a clearly defined AD-like phenotype, including Aβ accumulation, cognitive impairment, and synaptic dysfunction. This selection is restricted to models for which network dysfunction has been reported in the literature. The timeline of the progression of the AD phenotype in each of the models is represented by Figure 2.

**Table 1.** Progression of AD pathology phenotype reported in several rodent models of AD, as presented in Figure 2.

Animal model	Soluble Aβ	Insoluble Aβ	Synaptic loss	Gliosis	Synaptic dysfunction	Cognitive impairment	Neuronal loss	References
APP/PS1 mice	*Not reported*	1.5 months	1 month	1.5 months	8/10 months	6 months	17 months	^174,183–186^
5xFAD mice	1.5 months	2 months	4 months	2 months	4 months	4 months	9 months	^176,187–189^
3xTg-AD mice	*Not reported*	6 months	3 months	7 months	6 months	4 months	*Not reported*	^177,190–192^
APP ^NL−F^ mice	2 months	6 months	9/12 months	6 months	*Not reported*	18 months	*Absent in the model*	^180^
APP ^NL−G−F^ mice	*Not reported*	2 months	4 months	2 months	3 months	6 months	*Absent in the model*	^179,193^
TgF344-AD rats	6 months	6 months	*Not reported*	6 months	6 months	6 months	16 months	^182,194,195^
APP23/PS45 mice	2 months	3 months	*Not reported*	*Not reported*	4 months	6 months	*Not reported*	^175,196^
Tg2576 (APPSwe)	6–8 months	11–13 months	4.5 months	10–16 months	5 months	6 months	Absent in the model	^178,197–202^

## Network dysfunction and synaptic plasticity

In vivo experiments in APP/PS1 mice analyzing LTP (high frequency stimulation) in the CA1 area of the hippocampus found LTP to decay with time in mice with Aβ plaques in the hippocampus.^185^ Similarly, ex vivo recordings in hippocampal slices of APP/PS1 mice showed CA1 pyramidal cells to display impaired spike timing-dependent plasticity as studied through LTP protocols induced by theta bursts in the Shaffer collateral.^203^ This impairment correlated with the proximity of the recorded neuron to an Aβ plaque.^203^ LTP induced at the Schaffer collateral and recorded extracellularly in the CA1 region in ex vivo hippocampal slices of adult Tg2576 mice was abolished, while LTP recorded in CA3 upon mossy fiber stimulation was preserved and even enhanced.^204^ In aged Tg2576 mice, LTP recorded in CA1 reemerged but was not subject to depotentiation, coinciding with the selective loss of PV interneurons and most likely caused by disrupted NRG1/ErbB4 signalling.^205^ Electrophysiological studies with multi-electrode arrays in 5xFAD transgenic mice at a stage with Aβ plaques in the cortex and hippocampus show similarly marked impairments, where biphasic current pulses were simultaneously applied on ex vivo preparations in the Schaffer collateral of the hippocampus and the cortex.^187^ Measurements performed in the CA1 of the hippocampus and layer 5 of the somatosensory cortex showed basal transmission being reduced in both areas, and short-term plasticity not being affected in either.^187^ However, where theta burst stimulation LTP protocols were applied in both areas, layer 5 somatosensory neurons showed higher impairment compared to hippocampal neurons.^187^ Since theta burst stimulation LTP requires functioning NMDA receptors and calcium influx, the changes visible in these models could be explained by the previously described effects of AβOs on glutamatergic transmission.^206^

When studying the activity of CA1 pyramidal neurons using whole-cell patch clamp electrophysiology in hippocampal slices, 5xFAD mice with mild behavioral impairment and moderate Aβ plaque load showed a decrease in the amplitude and frequency of miniature inhibitory postsynaptic currents (mIPSC), an increase in the amplitude of miniature excitatory postsynaptic currents (mEPSC) together with a decrease in mEPSC frequency and an increase in intrinsic excitability of these neurons, indicating impaired inhibitory and excitatory communication.^207^ A possible role of inhibitory dysfunction underlying these findings was further supported by a hippocampal decrease in the ratio of α1-GABA_A_R/α3-GABA_A_R in the hippocampus of 5xFAD mice, as analyzed through western blotting.^207^ In Tg2576 mice, whole-cell patch clamp recordings from dentate gyrus mossy cells in ex vivo slices revealed early hyperexcitability at one month of age, with increased spontaneous excitatory postsynaptic current (sEPSC) frequency, decreased spontaneous inhibitory postsynaptic current (sIPSC) frequency and amplitude, and altered intrinsic properties (lower rheobase, higher firing rates), indicating a shift in excitation-inhibition balance before plaque deposition or cognitive deficits.^208^ Granule cells showed increased frequencies of both sEPSCs and sIPSCs, but no changes in intrinsic excitability at this stage.^208^ These results indicate that disruption to the function of several individual cell types might underlie visible network level impairments.

Interestingly, several different effects have been described on the dysfunction of network activity in these models. In *App^NL−G−F^* mice, we found the desynchronization of hippocampal CA3 FSN to gamma oscillations in ex vivo recordings as the earliest functional impairment preceding Aβ plaque formation and in the presence of soluble Aβ_1−42._^136^ Moreover, in the same mouse model, the disruption to the activity of hippocampal FSN was revealed to correlate with microglia alterations and Aβ-plaque load.^137^ When assessed throughout the progression of Aβ pathology in *App^NL−G−F^* mice, the power of gamma oscillations in ex vivo hippocampal slices was also observed to decrease during the early phase of Aβ pathology progression.^137^ In addition, the *App^NL−G−F^* and the *App^NL−F^* mice show an increased glutamate release probability in the CA1 area of the hippocampus before the detection of plaques.^209^ Using the *App^NL−G−F^*, researchers found an LTP deficit in the prefrontal cortex and increased prefrontal-hippocampal network synchronicity evaluated by resting-state functional MRI in the early stages of the Aβ pathology.^210,211^ Additionally, in Tg2576 mice younger than four months, extracellular field recordings in ex vivo entorhinal cortex slices demonstrated stimulus-evoked repetitive field potentials and a greater response to reduced extracellular magnesium, indicating intrinsic network hyperexcitability and axonal conduction defects before plaque deposition onset.^212^

Disrupted power of gamma frequency oscillations is also present in the 5xFAD model at early, moderate, and advanced stages of Aβ pathology, where the power of hippocampal gamma oscillations is decreased while the frequency variance of the oscillation increased, as compared to wild-type mice.^213–215^ These findings show that Aβ pathology affects the brain globally, with changes to network activity being related to specific effects on cell types whose activity contributes to the corresponding band frequency ranges. The disruption to network dynamics was also tested with in vivo electrophysiological recordings. Age-dependent network activity disruptions have been reported in the APP/PS1 mice, where the power of in vivo recorded theta-band oscillatory activity (3–12 Hz) in the hippocampus declined with the age of the mice, a decrease that also correlated with Aβ-plaque load.^216^ Similarly, when assessed in the neocortex of anaesthetized 3xTg-AD mice with early-stage and advanced Aβ pathology, frequency-specific impairments were observed: slow oscillation activity (<1 Hz) was reduced in the older group, with greater cycle variability and lower firing rates; “down” states became significantly longer with age, while “up” states remained largely unaffected.^217^ Beta-gamma oscillations (15–100 Hz) also shifted with mouse age, and cortical excitability initially rose but later became increasingly inhibited, culminating in prolonged “up” states and reduced firing rates.^217^ These findings correspond to the functional phenotype visible in patients, where early-stage hyperexcitability is followed by late-stage hypo-excitability as the pathology progresses.^218^

Recordings in freely moving Tg2576 mice show network abnormalities from an early age, with increased susceptibility to induced seizures and spontaneous epileptiform activity detectable as early as 1.5 months, prior to the onset of memory deficits.^219,220^ Tg2576 mice show frequent interictal spikes, predominantly during REM sleep, and a phase-locked relationship between epileptic events and the trough and ascending phase of theta oscillations (4–12 Hz), indicating abnormal pyramidal cell activity.^220^ Moreover, Tg2576 mice at 1 month of age display high-frequency oscillations (HFOs; 250–500 Hz) in the dentate gyrus and cortex, which are most prominent during slow wave sleep and are indistinguishable from those observed in epilepsy models.^221^ HFOs are also associated with interictal spikes and seizures, and their occurrence does not worsen with age, suggesting that hyperexcitability and network hypersynchrony are early and persistent features of this model.^219,221^

Anaesthetized TgF344-AD transgenic rats with Aβ plaque load at different stages also exhibited an age-dependent effect on oscillatory activity, involving a reduction of theta-band power and the corresponding peak frequency.^222^ Additionally, theta-phase gamma-amplitude coupling was significantly weaker in older TgF344-AD rats, affecting both low (30–55 Hz) and high (65–95 Hz) gamma bands.^222^ When assessed in freely-moving TgF344-AD rats, neocortical theta- and gamma-band power were reduced compared to age-matched controls.^222^ Similarly, cortico-hippocampal and cortico-cortical connectivity was also impaired in TgF344-AD rats, as measured by reduced phase-locking values between frontal-occipital cortices and frontal cortex-hippocampus.^222^ Additionally, awake but motionless TgF344-AD rats showed seizure-like hypersynchronous high-voltage spindles in the frontal cortex.^222^ These results indicate impairment that not only affects network activity as a whole but also shows specific signatures that demonstrate dysfunction that affects synaptic integration and communication between brain regions. Similarly to previous studies, wide-field calcium imaging experiments in APP23/PS45 mice with advanced Aβ pathology revealed cortical slow-wave coherence to be significantly reduced.^223^ This dysfunction was also confirmed by electrophysiology, where hippocampal-cortical and cortico-thalamic slow-wave coherence was decreased in APP23/PS45 mice compared to wild type.^223^ The experimental paradigm also included topical exposure of the cortex of wild-type mice to synthetic mixed-form Aβ_1−40_ and Aβ_1−42_, which same as in APP23/PS45 disrupted slow-wave synchrony. This effect was rescued through a pharmacological intervention with midazolam, a positive allosteric modulator for GABA receptors, indicating impaired GABAergic transmission as underlying the dysfunction.^223^

Growing evidence across amyloid-producing mouse models indicates that Aβ disrupts synaptic and network function across brain regions in a sex-dependent manner. Tg2576 mice exhibit early, progressive, and sex-dependent cognitive deficits, including impaired water maze retention and circular platform performance, particularly in females at 3 months, and a progressive decline in males by 9 months, prior to overt amyloid deposition.^224^ More recently, APP/PS1 female mice were shown to have more pronounced hippocampal network dysrhythmia and aberrant excitability than males, suggesting that Aβ accumulation perturbs oscillatory coordination differently across sexes.^225^ Additionally, Aβ-driven alterations in excitation–inhibition balance and neuronal synchrony also diverge between males and females APP/PS1 mice, with females showing earlier and more severe impairments in synaptic function and circuit stability.^226^ This sex-dependent network vulnerability or resilience may help explain divergent trajectories of hippocampal dysfunction observed in male and female AD patients.^227,228^

Altogether, findings in AD animal models reveal widespread network dysfunction, with impaired synaptic plasticity, disrupted oscillatory activity, and weakened long-range connectivity. Aβ pathology alters excitatory-inhibitory balance, leading to early hyperexcitability and later large-scale network signaling impairment.

## Electrophysiological changes due to Aβ pathology in humans

Cognitively unimpaired individuals with elevated cerebral Aβ burden exhibit electroencephalographic (EEG) and magnetoencephalographic (MEG) alterations. Aβ deposition in cognitively unimpaired adults is predictive of a progressive slowing of oscillatory brain activity, particularly a shift toward lower-frequency theta and delta rhythms, accompanied by a gradual reduction in global functional connectivity.^229^ Additionally, in cognitively intact individuals, Aβ accumulation was distinctly associated with disrupted neuronal synchrony patterns measured via EEG and MEG, even before tau pathology or cognitive decline became evident.^230^ These subtle electrophysiological shifts occurring in the absence of clinical symptoms suggest latent network dysfunction as an early hallmark of preclinical AD pathology.

**Figure 3.**
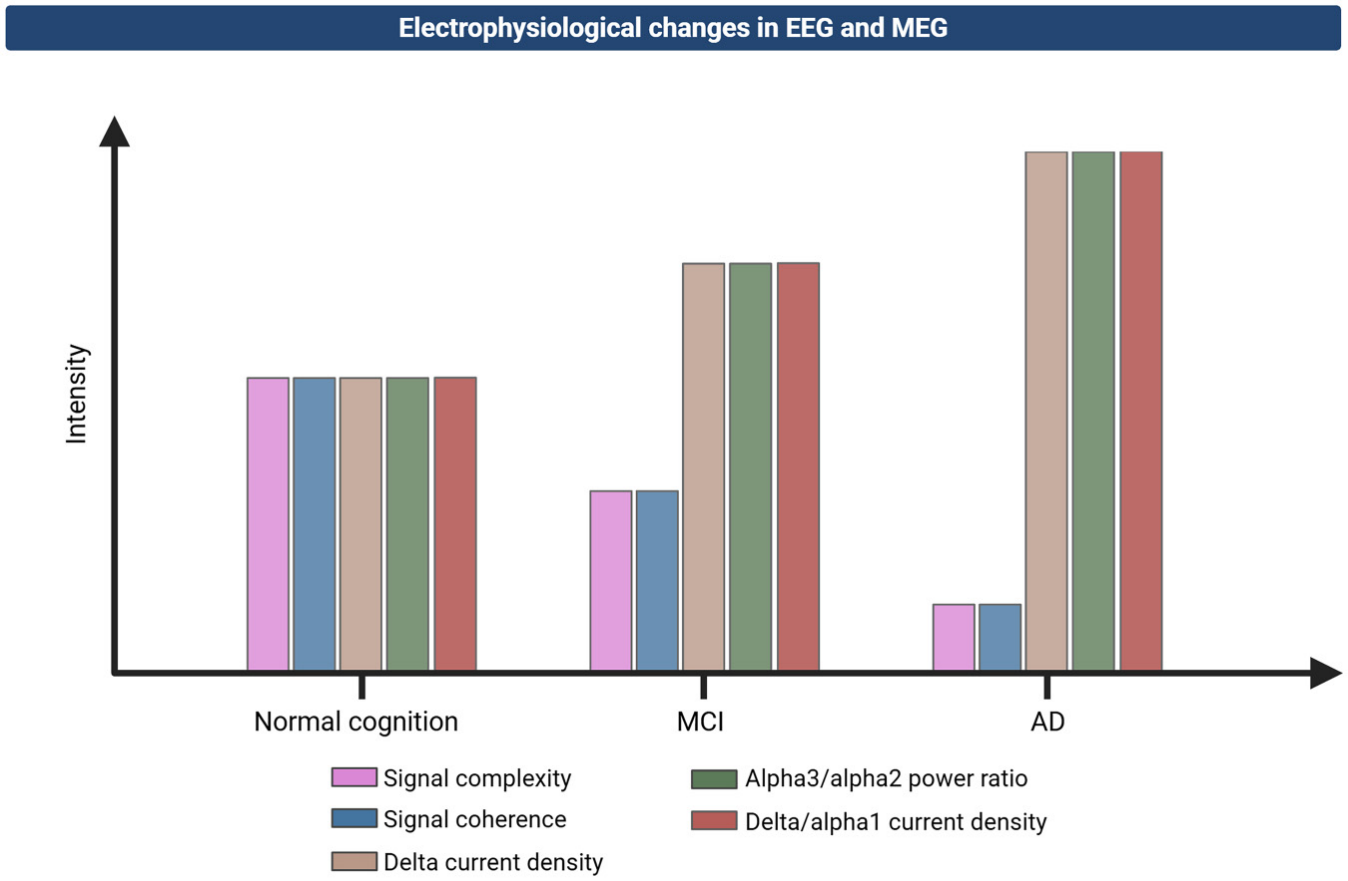
Electrophysiological changes in EEG and MEG between healthy, MCI, and AD patients. As mild cognitive impairment (MCI) appears, delta current density, delta/alpha1 current density and alpha3/alpha2 power ratios increase, signaling altered neuronal synchronization.^237^ Noticeable decreases are observed in signal complexity and coherence, indicating reduced network integration.^241,243,244^ By the AD stage, pronounced delta activity in right parietal cortex and precuneus, further increased delta/alpha1 current density and alpha3/alpha2 power ratios emphasize the cascading nature of synaptic and network-level dysfunction.^239,240,243,244^

Similar hallmarks can be found in the EEG and MEG readouts of individuals with cognitive impairment and AD. Those affected by AD show a pronounced shift of alpha rhythms from posterior to frontal brain areas.^231,232^ MEG analyses confirm that alpha amplitude often increases in these frontal regions, a pattern that is linked to poorer cognitive performance.^233,234^ By contrast, mild cognitive impairment (MCI) participants do not always display such a pronounced frontal alpha reorganization, and some investigations show that alpha generation remains predominantly posterior until further AD development.^235^ Additional studies indicate that the alpha peak frequency tends to slow in MCI, particularly in parieto-occipital areas, and that an elevated high-frequency alpha (alpha3) relative to low-frequency alpha (alpha2) power ratio is associated with increased cortical atrophy and a greater likelihood of eventual AD diagnosis, as visualized in disease progression in Figure 3.^236,237^ Hippocampal atrophy is also found to coincide with reduced alpha power, especially in parietal and occipital cortices, while parietal to frontal alpha and theta coupling declines as disease severity progresses.^238,239^ In parallel, studies using MEG recordings show that heightened delta current density in the right parietal lobe and precuneus predicts conversion from MCI to dementia.^240^ Another resting-state EEG marker, presented in Figure 3, is the ratio of delta to low-frequency alpha (alpha1) current density, which demonstrates utility in distinguishing AD from healthy ageing with a high classification rate.^241,242^

Other EEG/MEG markers have focused on complexity measures that include reductions in frontal, temporal, parietal, and occipital regions that indicate a lower degree of information exchange between neuronal circuits in MCI and AD, as shown in Figure 3.^243,245,246^ Researchers further observed that epoch-based entropy and Hjorth mobility analyses could reliably differentiate these patient groups from cognitively healthy individuals due to correlations with Aβ and tau pathology.^247^ As presented in Figure 3, functional connectivity investigations support these findings by showing widespread decreases in coherence across alpha, theta, and delta frequencies, often more pronounced in interhemispheric electrode pairs.^244,248,249^ Classifier models that incorporate coherence and synchrony features were noted to achieve good diagnostic accuracy in detecting AD.^250^ Moreover, carriers of the *APOE4* allele exhibited enhanced alpha and beta connectivity in right-hemisphere networks, which indicates an early compensatory response that diminishes with further disease progression.^251^ Finally, reductions in global field synchronization for alpha and beta bands were strongly associated with declining Aβ and increasing tau in CSF, underscoring the interplay between oscillatory changes and established AD biomarkers.^252^

Recently, a new non-invasive biomarker has been proposed based on pre-clinical studies, namely, high-frequency oscillations (HFOs).^221^ High-band HFOs (>250 Hz) were reported during non-REM sleep in wideband scalp EEG recordings from individuals with Down syndrome, before AD pathology, and appearing independently of epilepsy diagnosis.^253^ These HFOs also showed right-hemisphere dominance in patients without AD-related cognitive decline and less consistently in patients with AD-related cognitive decline.^253^ MEG recordings further revealed elevated ripples (80–250 Hz) and fast ripples (250–500 Hz) across multiple regions in AD patients, higher in non-epileptic than epileptic subtypes at baseline, alongside right-hemisphere asymmetry in epileptic AD patients.^254^ Though promising as a non-invasive hyperexcitability biomarker, more research is needed to understand the role of HFO in the progression of AD.^253,254^

## Discussion

Aβ plays a pivotal role in the pathogenesis of AD, contributing to dysfunction at multiple levels within the central nervous system. A general theme emerging from the literature is that Aβ exerts its toxic effects on functional activity by affecting multiple pathways that interconnect synaptic dysfunction, neurotransmitter imbalance, and calcium homeostasis. This destabilizes the function of the brain at several levels, ranging from single neurons, networks and large-scale brain activity.

### Roles of Aβ in synaptic neurotoxicity

Aβ-induced neurotoxicity begins at the molecular level, with AβOs, including protofibrils, identified as the most neurotoxic forms. Their soluble and diffusible nature allows them to interact with key synaptic receptors and proteins, disrupting calcium homeostasis, synaptic architecture, and mitochondrial function.^16,86,98,99,116,153^

The toxic impact of Aβ extends further to influence broader neurotransmitter systems. Aβ disrupts cholinergic signaling by targeting nAChRs, a key pathway for synaptic plasticity and cognitive function.^101,104,107^ Furthermore, the loss of cholinergic signaling precedes neuronal death, implicating this early dysfunction in AD progression.^104,108^ GABAergic neurons are sensitive to AβOs, which play a central role in the early loss of inhibitory control in AD.^48,126,135^ In both mouse models and the human cortex, Aβ pathology leads to a decrease in the number of GABAergic interneurons.^255,256^ Aβ also targets glutamatergic signaling pathways, disrupting excitatory/inhibitory balance and inducing pathological synaptic responses.^99,122,257^ Interactions with AMPA, NMDA and metabotropic glutamate receptors lead to structural damage, synapse loss, and impaired gamma oscillations.^50,99,136,137,257,258^ These findings underscore the interconnected nature of Aβ-induced damage across neuronal systems.

### From local damage to global network dysfunctions

Aβ pathology impairs synaptic plasticity, as shown by reduced LTP in hippocampal and cortical neurons, particularly near plaques.^203^ These changes likely reflect early disruption of NMDA receptor-dependent glutamatergic signalling,^206^ pointing to Aβ's direct interference with mechanisms essential for memory encoding.

At the cellular level, Aβ alters the balance of excitation and inhibition. In CA1 pyramidal neurons, inhibitory input is diminished, excitatory postsynaptic currents are modified, and intrinsic excitability is increased.^207^ These disturbances manifest in altered network dynamics. Gamma-band desynchronization appears early in disease models, affecting fast-spiking interneurons and pyramidal neurons.^136^ Reductions in gamma power and increased variability across models reflect impaired temporal precision in local networks.^216,217,222^ These changes are selective for certain cell types and frequency ranges, suggesting targeted vulnerability of oscillatory mechanisms to Aβ toxicity.

With disease progression, long-range coordination deteriorates. Decreased coherence in theta and slow-wave bands between hippocampus, cortex, and thalamus, along with increased spectral variability, signal large-scale disintegration of functional coupling.^216,217,222^ These dynamics resemble the clinical transition from early-stage hyperactivity to late-stage hypoexcitability.^218^ Partial restoration of synchrony by GABAergic modulation supports a central role for inhibitory network failure in this decline.^223^ Human studies support these findings. In AD, alpha rhythms shift anteriorly and increase in frontal amplitude, patterns associated with reduced cognitive performance.^231,233^

Advancing pathology is marked by reduced parietal-frontal coherence, elevated delta activity, and decreased signal complexity, reflecting breakdowns in integrative processing.^239,240,243,244^ These changes are more pronounced in *APOE4* carriers, who show an initial increase in connectivity that diminishes with further progression.^251^ Loss of synchrony in alpha and beta bands aligns with CSF markers of lower Aβ and higher tau, linking electrophysiological changes to molecular pathology.^252^

Together, these findings show that Aβ pathology destabilizes neuronal function across scales—molecular, cellular, and network—through progressive, layered disruption. Aβ acts not through a single target, but by undermining the architecture of brain activity itself.

### Broad perspective for disease-modifying treatments

These findings emphasize the need for therapeutic strategies targeting the multifaceted roles of Aβ in AD. Current Aβ-targeting therapies, including antibody-based approaches, show promise but often lead to mixed outcomes.^24,259,260^ One of the key concepts to highlight is the direct effects of Aβ on neuronal function and homeostasis, such as excitatory/inhibitory imbalance, and not by looking at metrics such as cognition, whose disruption results from a multitude of factors that, except for functional impairment, include tau pathology, neurodegeneration, and neuroinflammation.^18^

Crucially, consolidated research indicates that Aβ accumulation occurs well before the onset of irreversible damage,^18^ presenting an opportunity for early intervention. The emerging ability to detect elevated levels of soluble Aβ and its associated disruptions through non-invasive biomarkers, such as blood-based assays,^12,13^ opens the door for preclinical diagnosis and preventive treatments. Furthermore, early changes of neurophysiological signatures can be used as potential biomarkers to be a complementary strategy for early detection of AD.^261^ Recent translational research has emphasized that such biomarkers could form the basis of precision-guided preventive therapy, potentially enhancing treatment efficacy and delaying or halting disease progression in high-risk populations.^262^

With this review, we encourage the field to consider Aβ's dual role in physiology and pathology for diagnosis and treatments. Combination therapies that address both Aβ accumulation and its downstream effects on synaptic integrity may offer a more comprehensive approach to preserving cognitive function. Moreover, identifying high concentrations of soluble Aβ as neurotoxic and recognizing its downstream effects as critical steps in disease progression will be essential for the accurate evaluation of Aβ therapies and their impact on AD. By integrating early detection with targeted therapeutic strategies, we may shift the paradigm from symptom management to true disease prevention.

## Conclusion

Aβ's role in AD extends beyond its accumulation into plaques; it initiates a cascade of dysfunction affecting synaptic transmission, intracellular signaling, and brain network activity. These findings highlight the complexity of Aβ's involvement in AD pathology, where its physiological functions become pathological in prodromal stages of the disease. Therefore, we hypothesize that synaptic toxicity could serve both as a biomarker and a therapeutic target for the preventive treatment of AD. As our knowledge of Aβ biology deepens, so will our capacity to design targeted interventions that address the root causes of AD.
